# Comparative Pharmacological Assessment of Amoxicillin in Five Cultured Fish Species: Implications for Off-Label Use in Aquaculture

**DOI:** 10.3390/antibiotics14040346

**Published:** 2025-03-27

**Authors:** Jun Sung Bae, Chae Won Lee, Chan Yeong Yang, Eun Ha Jeong, Bosung Kim, Kwan Ha Park, Jung Soo Seo, Mun-Gyeong Kwon, Ji-Hoon Lee

**Affiliations:** 1 Department of Aquatic Life Medicine, Kunsan National University, Gunsan 54150, Republic of Korea; 1301806@kunsan.ac.kr (J.S.B.); chaeee1@kunsan.ac.kr (C.W.L.); misha9703@kunsan.ac.kr (C.Y.Y.); dmsgk1532@kunsan.ac.kr (E.H.J.); fishpath@kunsan.ac.kr (B.K.); khpark@kunsan.ac.kr (K.H.P.); 2 Aquatic Disease Control Division, National Fisheries Products Quality Management Service, 337 Haeyang-ro, Yeongdo-gu, Busan 49111, Republic of Korea; jsseosoo@korea.kr (J.S.S.); mgkwon@korea.kr (M.-G.K.)

**Keywords:** amoxicillin (AMOX), antibacterial efficacy, major aquaculture species, residue depletion, withdrawal time

## Abstract

**Background**: Amoxicillin (AMOX) is widely used in aquaculture for bacterial infections due to its efficacy and safety. Despite official approval for select species, off-label use is common. This study evaluated the antibacterial efficacy and residue depletion of AMOX in five aquaculture species: olive flounder (*Paralichthys olivaceus*), rainbow trout (*Oncorhynchus mykiss*), Japanese eel (*Anguilla japonica*), black rockfish (*Sebastes schlegelii*), and Israeli carp (*Cyprinus carpio*). **Methods**: Fish were administered AMOX orally at 40 mg/kg and 80 mg/kg for seven days. Antibacterial efficacy was assessed by bacterial load reduction and survival rates following artificial infection. Residue depletion was analyzed using HPLC–MS/MS to determine the time required for AMOX levels to fall below the maximum residue limit (MRL, 0.05 mg/kg). **Results**: AMOX, at 40 mg/kg, significantly reduced bacterial loads in olive flounder, rainbow trout, and Japanese eel (*p* < 0.05), while Israeli carp exhibited a limited response (*p* = 0.54). Black rockfish showed moderate efficacy (RPS 72.7%) but increased mortality at 80 mg/kg. Residue levels fell below the MRL within 10 days for all species except Israeli carp (~30 days). **Conclusions**: These findings highlight species-specific differences in AMOX efficacy and residue depletion rates, emphasizing the necessity of tailored dosing regimens and withdrawal periods to ensure optimal therapeutic outcomes and food safety compliance in aquaculture. Further pharmacokinetic studies are needed to refine dosing strategies, particularly for species with extended residue retention and potential dose-dependent adverse effects.

## 1. Introduction

Amoxicillin (AMOX) is a semi-synthetic antibiotic derived from natural penicillin, designed to enhance its antibacterial spectrum and stability in gastric environments [1,2]. With robust activity, particularly against Gram-negative bacteria, AMOX has been widely applied in human and veterinary medicine to manage bacterial infections [3]. Its extensive use in aquaculture is also well-documented, targeting various bacterial diseases, including streptococcosis, furunculosis, pasteurellosis, and edwardsiellosis [4].

In countries like Korea and Japan, AMOX is officially approved for specific fish species, such as olive flounder (*Paralichthys olivaceus*) and yellowtail (*Seriola quinqueradiata*), for the treatment of streptococcosis and pseudotuberculosis, respectively [5,6]. Although injection is a common administration route for antibiotics in aquaculture, achieving significant therapeutic outcomes [7,8,9,10], this method is associated with risks such as tissue damage, inflammation, and necrosis at the injection site [11,12,13,14,15]. Recovery at the injection site may require extended periods, making oral administration a more practical and less invasive alternative for commercial farming [16].

Oral AMOX administration is especially preferred for adult fish due to its shorter withdrawal period and rapid residue depletion from muscle tissue [17,18]. For example, in Korea, the maximum residue limit (MRL) for AMOX is set at 0.05 mg/kg, and the withdrawal period for yellowtail is established as 7 days when administered orally at 40 mg/kg for 7 consecutive days [6]. However, yellowtail represents only 5% of the total aquaculture production in Korea [19]. In contrast, black rockfish (*Sebastes schlegelii*), olive flounder, Israeli carp (*Cyprinus carpio*), Japanese eel (*Anguilla japonica*), and rainbow trout (*Oncorhynchus mykiss*) are industrially significant, contributing over 60% of the country’s aquaculture production in 2022 [19].

Despite its widespread use, AMOX is often administered off-label, relying on expert judgment without official authorization for several species, resulting in limited data on efficacy and withdrawal periods [20]. While some studies have estimated withdrawal periods for olive flounder in farm conditions, critical factors such as water temperature variations under controlled experimental settings remain unexplored.

In this context, the present study investigated the antibacterial efficacy and residue depletion of AMOX following a 7-day oral administration regimen at 40 mg/kg in black rockfish, olive flounder, Israeli carp, Japanese eel, and rainbow trout. Unlike previous studies that focused on individual species, this study provides a comparative pharmacological assessment across multiple commercially important fish species, offering new insights into species-specific efficacy, residue depletion patterns, and withdrawal periods. By establishing withdrawal periods for these major cultured species in Korea, this research not only addresses the current gap in data for off-label AMOX use but also provides a scientific basis for optimizing AMOX administration strategies in aquaculture, ensuring both therapeutic effectiveness and compliance with food safety regulations.

## 2. Results

### 2.1. Comparative In Vivo Efficacy of AMOX in Artificially Infected Fish

**Figure 1:**
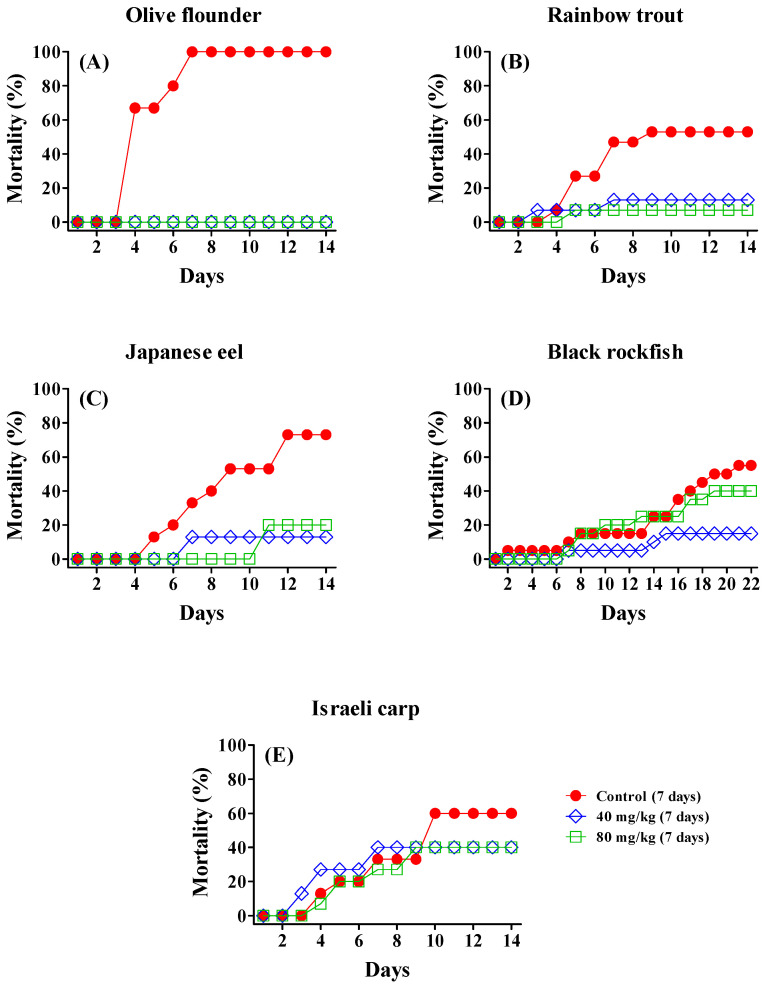
Effect of AMOX on cumulative mortality in fish artificially infected with bacterial pathogens. Fish were artificially infected with bacterial pathogens and subsequently administered AMOX orally for seven consecutive days. Cumulative mortality was monitored daily for up to 22 days post-infection. Each panel illustrates the cumulative mortality for a specific fish species: (**A**) olive flounder (n = 15), (**B**) rainbow trout (n = 15), (**C**) Japanese eel (n = 15), (**D**) black rockfish (n = 20), and (**E**) Israeli carp (n = 15).

The in vivo efficacy of AMOX was evaluated following a 7-day oral administration at doses of 40 and 80 mg/kg. AMOX was administered 24 h after artificial bacterial infection, and cumulative mortality was monitored for 14 days post-infection. The results are summarized in Table 1 and Figure 1.

In the control groups, mortality was observed between 2 and 5 days post-infection in all fish species, with cumulative mortality reaching 53.3% to 100.0% over 14 days. In contrast, groups treated with 40 mg/kg of AMOX exhibited significantly lower mortality in all species except Israeli carp, achieving relative percentage survival (RPS) values of 72.7% to 100.0% (*p* < 0.05). Olive flounder demonstrated complete protection with 100% survival, whereas rainbow trout and Japanese eel also showed substantial survival benefits with RPS values of 75.5% and 82.2%, respectively. Black rockfish exhibited moderate protection with an RPS of 72.7%. Israeli carp, however, showed partial protection with an RPS of 33.3%, but this result was not statistically significant (*p* = 0.5369).

Increasing the AMOX dose to 80 mg/kg did not yield significant improvements in mortality reduction for olive flounder, rainbow trout, or Japanese eel compared to the 40 mg/kg dose. Interestingly, black rockfish treated with 80 mg/kg AMOX experienced a higher mortality rate than the 40 mg/kg group, with an RPS decreasing to 27.3%, suggesting potential adverse effects at higher doses. These findings highlight species-specific differences in AMOX efficacy and dose response.

### 2.2. Species-Specific Residue Depletion and Withdrawal Periods of AMOX in Fish

The residue levels of AMOX in fish muscles were evaluated following a 7-day oral administration at doses of 40 and 80 mg/kg, as shown in Table 2. AMOX exhibited low residue levels and was rapidly eliminated from muscle tissues in most species, though interspecies differences were evident.

At a dose of 40 mg/kg under optimal temperature conditions, olive flounder, rainbow trout, and black rockfish displayed initial residue levels of 514 ± 571 µg/kg, 347 ± 362 µg/kg, and 298 ± 909 µg/kg, respectively, on day 1. These residues fell below the limit of quantification (LOQ) by day 7. Japanese eel showed a residue level of 49 ± 92 µg/kg on day 3, also dropping below the LOQ by day 7. In contrast, Israeli carp exhibited a slower elimination pattern, with residue levels of 289 ± 283 µg/kg on day 3 and 52 ± 34 µg/kg on day 7, requiring up to 28 days to fall below the LOQ.

At a dose of 80 mg/kg under optimal temperature conditions, residue levels were elevated across all species but followed similar elimination patterns. Olive flounder, rainbow trout, Japanese eel, and black rockfish showed residues below the LOQ by day 14. However, Israeli carp required up to 42 days to reach below the LOQ, reflecting its significantly slower elimination kinetics compared with other species.

Under suboptimal temperature conditions, the residue levels in olive flounder, rainbow trout, and black rockfish were slightly lower than those under optimal conditions. On day 1, their residue levels were 222 ± 279 µg/kg, 239 ± 273 µg/kg, and 116 ± 148 µg/kg, respectively, and residues fell below the LOQ by day 7, showing a similarly rapid elimination pattern. Japanese eel and Israeli carp, regardless of temperature conditions, followed elimination patterns comparable to those under optimal conditions, with residues falling below the LOQ after 14 and 42 days, respectively.

Withdrawal periods were calculated using an MRL of 0.05 mg/kg, as presented in Table 3. The withdrawal periods for AMOX following a 7-day oral administration were determined to be 6.1 to 6.6 days for olive flounder, 7.0 to 7.8 days for rainbow trout, 9.4 to 16.3 days for Japanese eel, 6.4 to 11.5 days for black rockfish, and 29.1 to 33.5 days for Israeli carp. These findings highlight significant interspecies variability in AMOX residue depletion rates, emphasizing the necessity of species-specific withdrawal period recommendations. Such data are critical for ensuring compliance with international food safety standards in aquaculture practices.

## 3. Discussion

This study was designed to demonstrate the broader utility of amoxicillin (AMOX), a semi-synthetic antibiotic widely used across various species globally [21,22], in additional fish species that have yet to be verified for efficacy and safe withdrawal times. Protective effects against respective bacterial pathogens and residue clearance patterns were examined in five commercially significant aquaculture species: olive flounder (*Paralichthys olivaceus*), rainbow trout (*Oncorhynchus mykiss*), Japanese eel (*Anguilla japonica*), black rockfish (*Sebastes schlegelii*), and Israeli carp (*Cyprinus carpio*).

In most countries, the limited number of aquaculture drugs officially marketed for a few fish species results in the frequent off-label use of these drugs in unapproved species [23,24]. The findings of this study emphasize the importance of generating correct and comprehensive information on drug efficacy and withdrawal periods for unapproved species to ensure their legitimate utility in aquaculture practices. Such data are invaluable for promoting therapeutic success while maintaining compliance with food safety standards.

Differences in AMOX efficacy among fish species are directly linked to variations in tissue drug levels, particularly concentrations in the blood after administration [25,26]. Tissue levels reflect the absorption rates of AMOX, which vary significantly between species. Previous studies have reported very low bioavailability in Japanese eel (≤1.6%) and red seabream (0.33%) when administered at 40 mg/kg, while olive flounder exhibited relatively higher bioavailability (9%) at the same dose [27,28,29]. These differences in absorption influence the therapeutic effectiveness of AMOX, particularly for time-dependent antibiotics such as beta-lactams, which rely on maintaining blood concentrations above the minimum inhibitory concentration (MIC) for at least 12 h to exert their antibacterial effects [30,31].

Interestingly, despite an increased dose of 80 mg/kg, the therapeutic efficacy of AMOX in this study did not significantly improve compared to 40 mg/kg, a result consistent with prior findings [28,29]. This suggests that higher doses may not proportionally enhance blood concentrations or therapeutic outcomes, particularly in species like Japanese eel and olive flounder, where rapid drug excretion limits prolonged therapeutic effects [32,33].

Moreover, in black rockfish, treatment with 80 mg/kg AMOX resulted in a higher mortality rate compared with the 40 mg/kg group, with relative percent survival (RPS) decreasing to 27.3%. Although direct evidence of AMOX toxicity in black rockfish is unavailable, the observed dose-dependent increase in mortality suggests that higher doses may exceed the species’ metabolic or excretory capacities, potentially leading to tissue-specific drug accumulation or toxic effects.

While muscle residues were used in this study as an indirect measure of absorption, direct blood concentration data (e.g., Cmax) would provide a more precise understanding of interspecies variability in bioavailability and efficacy. Furthermore, such data could clarify whether higher doses exacerbate toxicity without proportionally improving therapeutic outcomes in species like black rockfish.

Residue depletion analysis revealed significant interspecies differences in the pharmacokinetics of AMOX. Olive flounder, rainbow trout, Japanese eel, and black rockfish exhibited rapid elimination of AMOX residues from muscle tissues, with residue levels falling below the maximum residue limit (MRL, 0.05 mg/kg) within 7–14 days after oral administration at 40 mg/kg. On day 1, residue levels reached 514 µg/kg, 347 µg/kg, and 298 µg/kg in olive flounder, rainbow trout, and black rockfish, respectively, before rapidly declining to the LOQ (<5–10 µg/kg) by day 7. Japanese eel also exhibited residues at the MRL level (49 µg/kg) on day 3, with most individuals falling below detection limits by day 7. Even when the dosage was increased to 80 mg/kg, residues in these four species remained well below the MRL by day 7, indicating their safety for consumption [20,28].

In stark contrast, Israeli carp demonstrated slower residue depletion, with MRL-level residues (52 µg/kg) on day 7, becoming below the LOQ only by day 28. At a higher dose of 80 mg/kg, residues in Israeli carp exceeded the MRL by approximately fivefold (273 µg/kg) on day 7 and required up to 42 days to decrease to below the LOQ. This extended withdrawal period is consistent with observations in fish with reduced metabolic activity and slower excretory processes [34,35,36].

Water temperature is a key factor influencing drug metabolism and elimination in fish. Previous studies have shown that fish metabolism and excretion rates decrease by approximately 10% for every 1 °C drop in water temperature [34]. In this study, AMOX withdrawal periods were consistent across most species under optimal water temperature conditions, with olive flounder, rainbow trout, and black rockfish requiring 6.1–7.4 days. However, Japanese eel showed a prolonged withdrawal period under suboptimal conditions (16.3 days at 20 °C), reflecting reduced metabolic and excretory rates. Israeli carp exhibited negligible differences in withdrawal periods between optimal (29.2 days) and suboptimal (29.1 days) conditions, suggesting inherently slow metabolism largely unaffected by temperature changes. Interestingly, rainbow trout showed a slightly longer withdrawal period under suboptimal conditions, likely due to increased drug absorption. In contrast, black rockfish exhibited faster clearance under suboptimal conditions, indicating metabolic adaptability.

These findings are consistent with earlier studies reporting rapid residue elimination in olive flounder, rainbow trout, and Japanese eel under optimal conditions but prolonged withdrawal periods in slower-metabolizing species like Israeli carp [18,37,38].

The prolonged residue elimination in Israeli carp can be primarily attributed to its unique gastrointestinal anatomy. Unlike olive flounder, rainbow trout, or black rockfish, Israeli carp, a member of the Cyprinidae family, lacks a functional stomach. This anatomical trait has been reported to result in slower rates of nutrient absorption, digestion, and excretion. Ref. [39] demonstrated that fish species with a stomach, such as striped catfish, exhibited faster drug metabolism compared with stomach-less species like grass carp. Similarly, ref. [40] reported that tiger puffer (*Takifugu rubripes*), which lacks a stomach, showed delayed excretion rates compared with red sea bream (*Pagrus major*), a species with a stomach.

Recent studies have also highlighted species-specific variations in AMOX residue depletion across different aquaculture species. For instance, in Nile tilapia (*Oreochromis niloticus*), withdrawal periods of 6 days and 4 days were observed at 25 °C and 30 °C, respectively, following oral administration of 40 mg/kg for 5 days [41]. Similarly, in striped catfish (*Pangasianodon hypophthalmus*), a withdrawal period of just 11 h was reported at 30 °C with a dosage of 50 mg/kg for 5 days [42]. These findings suggest that higher water temperatures can significantly accelerate drug elimination, potentially due to increased metabolic rates at elevated temperatures.

Comparatively, the withdrawal periods observed in this study were longer than those reported for Nile tilapia and striped catfish, which may be attributed to differences in species-specific drug metabolism, gastrointestinal physiology, and excretory capacity. These results reinforce the importance of evaluating AMOX residue depletion in a species-specific manner to ensure accurate withdrawal period determinations and food safety compliance in aquaculture.

The reliance on off-label AMOX use due to the limited availability of approved drugs highlights the necessity of robust pharmacokinetic and pharmacodynamic (PK/PD) data. Fish species such as Israeli carp, which lack a functional stomach, exhibit unique metabolic profiles that may influence drug absorption and elimination [37]. Without such data, optimizing dosing regimens and predicting withdrawal periods remains challenging, increasing the risk of non-compliance with international food safety standards.

This study provides valuable insights into the broader application of AMOX in aquaculture, demonstrating its therapeutic potential in species such as olive flounder, rainbow trout, and Japanese eel. However, the limited efficacy in Israeli carp and the prolonged residue persistence necessitate further investigation into pharmacokinetics, minimum inhibitory concentrations (MICs), and alternative treatment strategies. Correct and comprehensive data on unapproved species are essential for enabling safe and effective AMOX use, supporting both therapeutic success and compliance with food safety regulations.

## 4. Materials and Methods

### 4.1. Chemicals and Reagents

The test drug, amoxicillin trihydrate (purity ≥ 98%), used in this study, was procured from TCI (Tokyo, Japan). For the extraction of amoxicillin from fish tissues during HPLC–MS/MS analysis, HLB cartridges (500 mg, 6 cc; Oasis^®^, Waters, Milford, MA, USA) were employed. The microbial media utilized for bacterial culture and growth included brain–heart infusion broth (BHI), and brain–heart infusion agar (BHIA), all purchased from Difco (Sparks, MD, USA). Additional key reagents, such as carboxymethyl cellulose sodium (CMC), formalin, formic acid, and tricaine methanesulfonate (MS-222), were sourced from Sigma-Aldrich (St. Louis, MO, USA).

### 4.2. Fish Species and Experimental Conditions

Five fish species were obtained from aquaculture facilities in three different provinces in Republic of Korea. The sizes of the fish were as follows: olive flounder (90–110 g), rainbow trout (30–50 g), Japanese eel (100–250 g), black rockfish (50–80 g), and Israeli carp (50–65 g). The fish were acclimated under laboratory conditions for two weeks in approximately 700 L recirculating tanks (1.0 m × 0.8 m × 0.6 m) at a density of 15–20 fish per tank.

Water quality parameters were monitored daily at 10:00 AM using a YSI 556MPS multimeter (YSI, Yellow Springs, OH, USA) to maintain dissolved oxygen levels between 6 and 8 mg/L, pH levels between 7 and 8, and salinity levels between 28 and 32 psu for olive flounder and black rockfish. Water temperatures were maintained at species-specific optimal levels: 22 ± 3 °C for olive flounder, 15 ± 2 °C for rainbow trout, 28 ± 3 °C for Japanese eel, 15 ± 2 °C for black rockfish, and 25 ± 2 °C for Israeli carp.

In some tests, certain fish were subjected to reference temperatures to evaluate residue clearance times under suboptimal excretory conditions, with these temperature settings determined based on species-specific physiological and metabolic characteristics. For olive flounder, Japanese eel, and Israeli carp, sub-optimal temperatures were set lower than their optimal conditions, as these species exhibit reduced metabolic activity and slower drug clearance at lower temperatures. Conversely, for rainbow trout and black rockfish, sub-optimal temperatures were set higher, as these species naturally thrive in colder waters and experience increased metabolic stress at elevated temperatures, which could influence drug elimination rates. This approach aligned with established physiological principles and regulatory considerations in aquaculture research, as outlined in the guidelines of the National Institute of Fisheries Science [43].

All experimental procedures involving fish were conducted in compliance with the ethical guidelines of Kunsan National University and were approved by the Institutional Animal Care and Use Committee (IACUC) of Kunsan National University (Approval Code: No. 2022-1). Sample collection was performed in accordance with institutional and national regulations to ensure ethical treatment and welfare of the experimental animals.

### 4.3. Oral Administration of AMOX

AMOX was administered orally to fish for 7 consecutive days at doses of 40–80 mg/kg, with dosing performed daily at 10:00 AM. The drug was prepared as a suspension using 1% sodium carboxymethyl cellulose (Na-CMC) mixed with red edible food dye (Littes, Gyeonggi, Republic of Korea). A 1 mL disposable syringe fitted with an oral gavage needle was used to deliver 0.1 mL of the suspension directly into the stomach of each fish.

Prior to drug administration, fish were lightly anesthetized with tricaine methanesulfonate (MS-222; 50–200 mg/L). After administration, fish were observed for 5 min, and any individuals that vomited during this period were excluded from the experiment [17,36].

### 4.4. In Vivo Efficacy of AMOX in Artificially Infected Fish

The efficacy of AMOX against major pathogenic bacterial infections was evaluated in fish infected with selected bacterial strains. *Streptococcus iniae* was used for olive flounder, rainbow trout, and Japanese eel, while *Lactococcus garvieae* was used for black rockfish. These Gram-positive pathogens were chosen based on their known pathogenicity to these fish species [44,45,46,47,48,49]. Additionally, *Edwardsiella piscicida*, a Gram-negative pathogen, was selected as the challenge strain for Israeli carp based on previous research findings [50]. All strains were expected to be susceptible to AMOX (Table 4).

For the in vivo efficacy evaluation, bacterial strains were cultured on BHI agar at 28 °C for 24 h. Cultures were centrifuged at 4000 rpm for 15 min to remove the supernatant, and bacterial suspensions were prepared using PBS. The suspensions were injected intraperitoneally at a volume of 0.1 mL per fish with the following bacterial concentrations: olive flounder, *S*. *iniae* 1.0 × 10^6^ CFU/fish; rainbow trout, *S*. *iniae* 8.2 × 10^6^ CFU/fish; Japanese eel, *S*. *iniae* 1.0 × 10^7^ CFU/fish; black rockfish, *L*. *garvieae* 4.4 × 10^7^ CFU/fish; and Israeli carp, *E*. *piscicida* 2.0 × 10^8^ CFU/fish. The mortality-causing doses for each strain were determined through preliminary tests.

Following bacterial infection, AMOX was administered orally at doses of 40 and 80 mg/kg for 7 consecutive days, starting 24 h post-infection. A positive control group included fish that received only bacterial infection and PBS. Fish were maintained at their optimal temperatures, and cumulative mortality rates were monitored over 14 days.

The sample size of 15–20 fish per group was selected based on practical considerations in experimental infection models for aquaculture species. Given the ethical and logistical constraints associated with large-scale fish infection studies, this sample size provides a feasible balance between minimizing animal distress and obtaining statistically meaningful efficacy data. Moreover, bacterial infection trials in fish typically result in high infection rates and well-defined mortality patterns, enabling clear differentiation between treatment effects. This approach allowed for robust statistical evaluation of AMOX efficacy within the given sample size.

### 4.5. Residue Depletion of AMOX in Fish Under Optimal and Suboptimal Conditions

The depletion of AMOX residues was evaluated following oral administration at doses of 40–80 mg/kg for 7 consecutive days in five different fish species. The study was conducted at both optimal and suboptimal temperatures. Optimal temperature represents the conditions for maximum growth and metabolism, where drug depletion is expected to occur at the fastest rate. In contrast, suboptimal temperature was chosen near the tolerance limit, where fish can survive but with slower physiological processes.

Muscle tissue samples were collected at predetermined intervals after the completion of the 7-day oral administration. Sampling was conducted on days 1, 3, 7, 14, 28, and 42, with 15 fish sampled at each time point. For residue analysis, the entire muscle tissue from each fish was homogenized. A 2 g portion of the homogenized sample was used for AMOX extraction using an acetonitrile–water solution (80:20, *v*/*v*), following the method specified in the Korean Food Standards Codex [51]. Lipids were removed using hexane, and the extract was further purified by solid-phase extraction (SPE) with HLB cartridges.

Residue levels of AMOX in muscle tissues were quantified using LC–MS/MS. The final extract (2.5 mL) was filtered through a 0.2 µm polyvinylidene fluoride membrane filter before injection into the LC–MS/MS system.

### 4.6. HPLC–MS/MS Analysis of AMOX Residues in Fish Muscle

HPLC–MS/MS analysis was conducted using a high-performance liquid chromatography system (QSight™ LX-50, PerkinElmer, Waltham, MA, USA) coupled with a mass spectrometer (QSight™ 420 MassSpec, PerkinElmer, Waltham, MA, USA). A reverse-phase C18 column (2.1 mm × 150 mm, 1.8 μm, Agilent, Santa Clara, CA, USA) was employed, maintained at 40 °C. The mobile phase consisted of 0.1% formic acid in water (A) and acetonitrile (B), with a flow rate of 0.2 mL/min. The gradient elution program for mobile phase A was as follows: 0.0–10.0 min, 90–50%; 15.0 min, 50–25%; 15.1 min, 25–90%; and 15.1–20.0 min, 90–90%.

AMOX was quantified in positive ion electrospray mode by monitoring the transition from m/z 366 to m/z 349. The analytical method was adapted for seafood analysis, with a limit of quantification (LOQ) of 0.005 mg/kg [51]. Muscle tissue samples from olive flounder, rainbow trout, Japanese eel, black rockfish, and Israeli carp were analyzed after spiking with trace amounts of AMOX (5–500 µg/kg) to validate the method. The linearity of the standard curves exhibited r^2^ values ≥ 0.98 across all fish species.

While the LOQ was 0.005 mg/kg for olive flounder, rainbow trout, Japanese eel, and Israeli carp, it was determined to be 0.01 mg/kg for black rockfish due to signal-to-noise (S/N) ratios of 10:1. The recovery of AMOX residues from pre-spiked muscle tissues, as determined by the current analytical method, is presented in Table 5.

### 4.7. Data Representation, Survival Analysis, and Withdrawal Period Estimation

All data are expressed as mean ± standard deviation. Reduction in mortality rates due to AMOX administration was analyzed using Kaplan–Meier survival curves, and statistical significance was determined using the log-rank test at *p* < 0.05 (GraphPad Prism, version 5.0, La Jolla, CA, USA).

Withdrawal periods were estimated using the withdrawal time calculation program (WT 1.4 version) [52], with a maximum residue limit (MRL) of 0.05 mg/kg [51]. Cut-off points were made from the upper 95% limit at a 95% confidence level. Residue levels below the limit of quantification (LOQ) were denoted as <LOQ, and these values were input as the LOQ value when calculating withdrawal periods.

## 5. Conclusions

This study highlights the species-specific efficacy and residue depletion patterns of amoxicillin (AMOX) in aquaculture, demonstrating strong antibacterial effects in olive flounder, rainbow trout, and Japanese eel, but limited therapeutic response in Israeli carp and moderate efficacy with dose-dependent adverse effects in black rockfish. Residue analysis revealed that most species achieved safe residue levels within 10 days, except Israeli carp, which required approximately 30 days. These findings underscore the need for tailored dosing regimens and withdrawal periods to ensure both therapeutic efficacy and food safety compliance. While this study focused on five commercially important fish species, AMOX is widely used across various aquaculture species without species-specific approval. Future studies should evaluate AMOX’s efficacy, residue depletion patterns, and withdrawal periods in additional fish species to further optimize treatment protocols. Expanding such research will contribute to establishing more comprehensive and evidence-based guidelines for responsible AMOX use in aquaculture.

## Figures and Tables

**Table 1:** In vivo efficacy of AMOX against artificial *S*. *iniae*, *L*. *garvieae*, and *E*. *piscicida* infection.

Fish Species	Test Groups	Mortality (%) (Dead Fish/Total Fish)	RPS ^a^ (%)	*p*-Value ^b^
Olive flounder	Control (7 days)	100.0 (15/15)	NA	NA
	40 mg/kg (7 days)	0.0 (00/15)	100.0	<0.0001
	80 mg/kg (7 days)	0.0 (00/15)	100.0	<0.0001
Rainbow trout	Control (7 days)	53.3 (8/15)	NA	NA
	40 mg/kg (7 days)	13.3 (2/15)	75.5	<0.05
	80 mg/kg (7 days)	6.7 (1/15)	86.8	<0.01
Japanese eel	Control (7 days)	73.3 (11/15)	NA	NA
	40 mg/kg (7 days)	13.3 (2/15)	82.2	<0.005
	80 mg/kg (7 days)	20.0 (3/15)	72.6	<0.005
Black rockfish	Control (7 days)	55.0 (11/20)	NA	NA
	40 mg/kg (7 days)	15.0 (3/20)	72.7	<0.05
	80 mg/kg (7 days)	40.0 (8/20)	27.3	0.4512
Israeli carp	Control (7 days)	60.0 (9/15)	NA	NA
	40 mg/kg (7 days)	40.0 (6/15)	33.3	0.5369
	80 mg/kg (7 days)	40.0 (6/15)	33.3	0.4720

NA, not applicable. ^a^ Relative percentage survival (% RPS) = [1 − (% test group mortality/% control mortality)] × 100. ^b^ Survival data were analyzed using Kaplan–Meier curves and the log-rank (Mantel–Cox) test.

**Table 2:** Concentration of AMOX in muscle (µg/kg) after seven consecutive days of oral administration of 40 and 80 mg/kg in different fishes at optimal and sub-optimal water temperatures.

Dosage (mg/kg) and Temperature ^a^	Days	Muscle Concentration (µg/kg)				
		Olive Flounder	Rainbow Trout	Japanese Eel	Black Rockfish	Israeli Carp
40 mg/kg at optimal temperature	1	514 ± 571 (15/15)	347 ± 362 (15/15)	-	298 ± 909 (13/15)	-
	3	41 ± 24 (15/15)	9 ± 17 (7/15)	49 ± 92 (11/15)	<LOQ	289 ± 283 (15/15)
	7	0 ± 1 (1/15)	<LOQ	<LOQ	<LOQ	52 ± 34 (15/15)
	14	<LOQ	1 ± 3 (1/15)	3 ± 8 (2/15)	<LOQ	15 ± 11 (12/15)
	28	<LOQ	<LOQ	<LOQ	<LOQ	<LOQ
	42	<LOQ	<LOQ	<LOQ	<LOQ	<LOQ
40 mg/kg at sub-optimal temperature	1	222 ± 279 (15/15)	239 ± 273 (15/15)	-	116 ± 148 (14/15)	-
	3	5 ± 7 (6/15)	76 ± 238 (9/15)	56 ± 108 (9/15)	<LOQ	248 ± 172 (15/15)
	7	<LOQ	3 ± 6 (4/15)	14 ± 33 (4/15)	<LOQ	162 ± 114 (15/15)
	14	<LOQ	<LOQ	3 ± 8 (2/15)	<LOQ	39 ± 19 (15/15)
	28	<LOQ	<LOQ	<LOQ	<LOQ	5 ± 8 (6/15)
	42	<LOQ	<LOQ	<LOQ	<LOQ	<LOQ
80 mg/kg at optimal temperature	1	1129 ± 953 (15/15)	746 ± 782 (15/15)	-	786 ± 2013 (10/15)	-
	3	80 ± 44 (14/15)	43 ± 90 (8/15)	69 ± 152 (9/15)	<LOQ	442 ± 366 (15/15)
	7	2 ± 5 (3/15)	2 ± 5 (3/15)	3 ± 4 (5/15)	<LOQ	273 ± 368 (15/15)
	14	<LOQ	<LOQ	1 ± 3 (1/15)	<LOQ	102 ± 75 (15/15)
	28	<LOQ	<LOQ	<LOQ	<LOQ	12 ± 17 (8/15)
	42	<LOQ	<LOQ	<LOQ	<LOQ	<LOQ

^a^ Optimal and sub-optimal temperatures were defined as follows: olive flounder (22 °C and 13 °C), rainbow trout (15 °C and 22 °C), Japanese eel (28 °C and 20 °C), black rockfish (15 °C and 22 °C), and Israeli carp (25 °C and 13 °C). Data are presented as mean ± SD (standard deviation). The number in the parenthesis denotes the individual fish with AMOX concentrations above the LOQ (5–10 µg/kg). n = 15 fish per group; “-”, not tested; LOQ, limit of quantification.

**Table 3:** Withdrawal time of AMOX in various fish species at different doses and water temperatures.

Fish Species	Withdrawal Time (Days)		
	Optimal Temperature		Sub-Optimal Temperature
	AMOX 40 mg/kg	AMOX 80 mg/kg	AMOX 40 mg/kg
Olive flounder	6.1 days (22 °C)	6.3 days (22 °C)	6.6 days (13 °C)
Rainbow trout	7.0 days (15 °C)	7.8 days (15 °C)	7.5 days (22 °C)
Japanese eel	9.4 days (28 °C)	10.5 days (28 °C)	16.3 days (20 °C)
Black rockfish	7.4 days (15 °C)	11.5 days (15 °C)	6.4 days (22 °C)
Israeli carp	29.2 days (25 °C)	33.5 days (25 °C)	29.1 days (13 °C)

Data are presented as mean ± SD (n = 15 per group). The statistical tolerance limit was calculated with 95% confidence. The maximum residue limit (MRL) applied in this study was the official level of 50 µg/kg.

**Table 4:** Bacterial strains, challenge doses, and references used for efficacy evaluation of AMOX treatment in five fish species.

Fish Species	Bacterial Strain	Strain Code	Challenge Dose (CFU/Fish)	Reference
Olive flounder	*Streptococcus iniae*	KCTC3657	1.0 × 10^6^	[46]
Rainbow trout	*S. iniae*	KCTC3657	8.2 × 10^6^	[44]
Japanese eel	*S. iniae*	KCTC3657	1.0 × 10^7^	[47,48,49]
Black rockfish	*Lactococcus garvieae*	NIFS-20FBLac0001	4.4 × 10^7^	[45]
Israeli carp	*Edwardsiella piscicida*	KCTC 12267	2.0 × 10^8^	[50]

Gram-positive bacterial strains (*Streptococcus iniae* and *Lactococcus garvieae*) were chosen based on their pathogenicity in the respective fish species, while *Edwardsiella piscicida*, a Gram-negative pathogen, was selected for Israeli carp.

**Table 5:** Validation parameters for HPLC–MS/MS analysis of AMOX in fish muscle.

Fish Species	Spiked Level (μg/kg)	Inter-Day (n = 3)		LOQ (μg/kg)
		Accuracy (% Recovery)	Precision (% CV)	
Olive flounder	5	83.8 ± 4.1	4.8	5
	50	84.7 ± 1.1	13.1	
Rainbow trout	5	75.8 ± 9.4	12.4	5
	50	85.7 ± 4.7	5.5	
Japanese eel	5	83.8 ± 5.8	6.9	5
	50	104.4 ± 17.2	16.5	
Black rockfish	10	75.2 ± 3.6	4.8	10
	100	85.1 ± 6.4	7.6	
Israeli carp	5	78.9 ± 4.0	5.0	5
	50	87.4 ± 5.8	6.6	

## Data Availability

The raw data supporting the conclusions of this article will be made available by the authors on request.
